# Prevalence and Risk Factors of Stroke Among Children With Sickle Cell Disease: A Retrospective Study at a Tertiary Care Center

**DOI:** 10.7759/cureus.41960

**Published:** 2023-07-16

**Authors:** Ghidaa Babeer, Danah Omran, Noor Bawahab, Raghad W Mohammed Hussain, Osama Muthaffar, Fatmah Alzahrani, Jumana A Shafei

**Affiliations:** 1 Adult Neurology, King Abdulaziz University Hospital, Jeddah, SAU; 2 Radiology, King Faisal Specialist Hospital and Research Centre, Jeddah, SAU; 3 Pediatrics, King Abdulaziz University Hospital, Jeddah, SAU; 4 Pediatrics, King Abdulaziz University Faculty of Medicine, Jeddah, SAU; 5 Medicine, King Abdulaziz University, Jeddah, SAU

**Keywords:** neurological complications, silent infarcts, pediatric ischemia, pediatric stroke, sickle cell disease

## Abstract

Background

Sickle cell disease (SCD) is a common autosomal recessive inherited hemoglobin disorder in many countries. Neurological complications are among the most disabling complications in SCD. Stroke and cerebral vasculopathy can lead to further neurological insult. Ischemic insults, stroke, and silent infarcts are preventable causes of morbidity and mortality in SCD patients. Understanding the epidemiology and characteristics of such patients will help to prevent complications.

Methodology

This is a retrospective study conducted in a tertiary care center in Saudi Arabia. Cases of SCD admitted to the pediatric ward between the years 2019 to 2023 were included in the study. Demographic data, clinical diagnosis, and frequency of prior admissions were collected. Brain imaging results were reviewed and included. Furthermore, the study assessed common risk factors leading to developing a stroke in SCD pediatric patients. Risk factors and clinical outcomes after stroke were also included.

Results

Eighty-one patients were enrolled. The mean age of stroke patients was 8.21±3.50 years while the mean age of non-stroke patients was 6.24±3.76 years. More than half of the patients were females in both the stroke (61.50%) and non-stroke groups (52.90%). Thirteen SCD patients (16%) were diagnosed with stroke. Previous history of stroke, high mean corpuscular volume (MCV), and low red blood cells count (RBC) were statistically significant risk factors for stroke (p<0.0001), (p<0.0001), (p<0.03), respectively.

Conclusion

Stroke is one of the most devastating complications of SCD. The prevalence of stroke among SCD patients in our study was 16%. Transcranial Doppler ultrasound screening is the most important predictor of stroke.

## Introduction

Introduction

Sickle cell disease (SCD) is an inherited monogenetic disorder due to a single base-pair point mutation in the 𝞫-globin gene resulting in the substitution of the amino acid valine for glutamic acid in the 𝞫-globin chain [1]. The populations most commonly affected by SCD originate from the Middle East, Sub-Saharan Africa, South and Central America, the Caribbean, India, and parts of the Mediterranean [2-3]. SCD is the leading cause of stroke among pediatric age group [4]. The predominant neurological manifestations of SCD include ischemic and hemorrhagic stroke, silent infarcts, chronic headaches, epilepsy, and cognitive impairment [2]. Repeated vaso-occlusive crises in different organs can result in neurological, locomotor, renal, and cardiorespiratory complications that severely impact patients' quality of life and survival [2, 5-6]. Cerebral vasculopathy associated with SCD is the cause for both silent cerebral infarct and overt stroke which ultimately leads to cognitive and neurological impairment [7].

Hemoglobin SS-associated pediatric stroke prevalence was 11% in the United States and France prior to systematic screening and intervention [8]. The Cooperative Study of Sickle Cell Disease (CSSCD), the largest US multicenter longitudinal observational study of complications of SCD, reported an overall prevalence of stroke of 3.75% in all patients with SCD [9-10]. Moreover, a meta-analysis from Africa concluded that stroke affected 4.2% of SCD patients [11-12]. A local study done in the southwestern province showed that the prevalence of stroke was 7.5% [13]. Regarding risk factors, patients with HbSS genotype comprise the majority of stroke cases among SCD patients [14]. The occurrence of acute chest syndrome among SCD patients is one of the clinical features associated with the development of stroke [15]. Data from a French study revealed that the serum lactate dehydrogenase (LDH) level and reticulocyte count were significant independent factors associated with stroke in multivariate analyses [16].

Nowadays, with the technological advance of the use of transcranial Doppler (TCD) cerebral blood flow velocity measurement, it is currently the standard of care for screening. Vascular narrowing can be detected if abnormally high blood flow in one or more major arteries was documented [17], leading to better prediction of stroke risk, allowing preventative treatment (i.e. long-term red cell transfusion program) prior to the first stroke [18]. It has been reported that SCD is a relatively common genetic disorder in the Middle East [19]. On the other hand, stroke is a preventable cause of morbidity and mortality in patients with SCD [20]. To the extent of our knowledge, data regarding stroke among children who suffer from SCD in Saudi Arabia, especially in the Western province, are scarce. Hence, our study aims to provide insight into stroke among pediatric patients who were diagnosed with SCD in King Abdulaziz University Hospital to better understand the risk of its development and aid in early prevention.

## Materials and methods

Ethical approval

This study was approved by the Unit of Biomedical Research of King Abdulaziz University Faculty of Medicine with reference number 925-21. The study also adhered to the guidelines outlined in the Declaration of Helsinki. All revealing data were masked and patients' data were kept private and anonymous.

Study design and sampling methodology

Following the Strengthening the Reporting of Observational Studies in Epidemiology (STROBE) guideline for retrospective studies [21], we conducted this retrospective study at King Abdulaziz Hospital University, Jeddah, Saudi Arabia, which is a teaching tertiary-care center that includes pediatric wards, inpatients as well as outpatient clinics, and day care units. Children who were younger than 14 years of age, known cases of SCD, and admitted to the pediatric ward between the years 2019 to 2023 were included in the study. Hemoglobin electrophoresis was the investigation of choice for the diagnosis of SCD. Stroke was defined as rapidly developing clinical signs of focal (or global) disturbance of cerebral function, with symptoms lasting 24 hours or longer or leading to death with no apparent cause other than of vascular origin as defined by the World Health Organization [22]. 

Acute chest syndrome was defined as an acute respiratory illness with fever and/or respiratory symptoms such as cough, dyspnea, tachypnea, or hypoxia requiring hospitalization [23]. A datasheet comprising demographic data (medical record number, name, sex, nationality) was used. Also, data about the clinical diagnosis, including age at admission, clinical diagnosis at admission, length of stay, and frequency of prior admissions, were collected. Moreover, data on the occurrence of stroke confirmed by brain imaging were included. Furthermore, this paper studied the common risk factors leading to stroke in SCD pediatric patients as previous history of stroke, previous history of acute chest syndrome, hemoglobin F fraction (HbF %), steady state count of white blood cells and red blood cells, hemoglobin level, platelet, hematocrit level, reticulocytes, levels of vitamin D, and lactate dehydrogenase. Additionally, the treatment and outcomes of stroke were also evaluated.

Data analysis

The statistical analysis was performed using the Statistical Package for Social Science (SPSS program for Windows, version 20, IBM Corp, Armonk, USA). Data were expressed as mean +/- standard deviation (minimum - maximum) or number (percentage) as appropriate. The difference between patients with and without stroke was made using the Chi-square test for non-parametric parameters and unpaired student “t” test for parametric parameters. P-value <0.05 was recognized as statistically significant.

## Results

A total of 81 patients were included in the study. The mean age of stroke patients was 8.21±3.50 years while the mean age of non-stroke patients was 6.24±3.76 years. More than half of the patients were females in both the stroke (61.50%) and non-stroke groups (52.90%). Thirteen SCD patients (16%) were diagnosed with stroke. All patients with stroke were diagnosed based on clinical and radiological findings of computed tomography (CT) and magnetic resonance imaging (MRI) of the brain. Amongst the 13 patients, 61.5% presented with stroke at admission while 38.5% of patients developed stroke during admission. Acute chest syndrome, vaso-occlusive crisis, sequestration crisis, and renal disease were the primary diagnoses in patients who developed stroke during admission.

The most common clinical diagnoses at admission among non-stroke patients were vaso-occlusive crisis (25%), followed by acute chest syndrome and hemolytic crisis (13.2%) as shown in (Table 1). Previous history of stroke, high mean corpuscular volume (MCV), and low red blood cells count (RBC) were statistically significant risk factors for stroke (Table 2). Regarding stroke characteristics, most of the patients had ischemic infarctions. The most common presentation of stroke was seizure (53.80%) followed by limb weakness (23.08%) and headache (15.40%). All patients received an exchange transfusion, and antiepileptic medications were given to those patients who presented with seizures. Motor sequelae were the most common outcomes among stroke patients (Table 3). Only six non-stroke patients had previous transcranial Doppler ultrasound screening (TCD). The results of CT and MRI brain are summarized in (Table 4).

**Table 1:** Demographic characteristics of the patients The asterisk sign (*) indicates a statistically significant relationship (p<0.05). SF: Sickle/Fetal; SS: Sickle/Sickle; SB: Sickle/Beta; SA: Sickle/Alpha

Characteristics	All patients (n= 81)	Patients with stroke (n =13)	Patients without stroke (n =68)	P-value
Age (years)	6.55±3.77 (0.33-13.00)	8.21±3.50 (0.67-13.00)	6.24±3.76 (0.33-13.00)	0.086
Gender	n (%)			0.398
Female	44 (54.30%)	8 (61.50%)	36 (52.90%)	
Male	37 (45.70%)	5(38.50%)	32 (47.10%)	
Nationality	n (%)			0.047*
Saudi	35 (43.20%)	4 (30.80%)	31 (45.60%)	
Non-Saudi	46 (56.80%)	9 (69.70%)	37 (54.40%)	
Clinical diagnosis at admission	n (%)			0.0001*
Vaso-occlusive crisis	18 (22.20%)	1 (7.70%)	17 (25.00%)	
Acute chest syndrome	11 (13.60%)	2 (15.40%)	9 (13.20%)	
Hemolytic crises	9 (11.10%)	0 (0.0%)	9 (13.20%)	
Stroke	8 (9.90%)	8 (61.50%)	0 (0.0%)	
Lower respiratory tract infections	8 (9.90%)	0 (0.0%)	8 (11.80%)	
Bone diseases	7 (8.60%)	0 (0.0%)	7 (10.30%)	
Upper respiratory tract infection	6 (7.40%)	0 (0.0%)	6 (8.80%)	
Gallbladder diseases	3 (3.70%)	0 (0.0%)	3 (4.40%)	
Sequestration crisis	2 (2.50%)	1 (7.70%)	2 (3.00%)	
Acute ductylitis	2 (2.50%)	0 (0.0%)	2 (2.90%)	
Aplastic crisis	1 (1.20%)	0 (0.0%)	1 (1.50%)	
Febrile convulsions	1 (1.20%)	0 (0.0%)	1 (1.50%)	
Headache	1 (1.20%)	0 (0.0%)	1 (1.50%)	
Renal diseases	2 (2.40%)	1 (7.70%)	1 (1.50%)	
Sepsis	1 (1.20%)	0 (0.0%)	1 (1.50%)	
Stuttering priapism	1 (1.20%)	0 (0.0%)	1 (1.50%)	
Length of stay (days)	11.69±12.76 (2.00-71.00)	21.85±20.49 (7.00-71.00)	9.96±9.77 (2.00-49.00)	0.002*
Confirmation with Hb electrophoresis	n (%)			0.368
SF	56 (69.10%)	7 (53.80%)	49 (72.10%)	
SS	22 (27.20%)	6 (46.20	16 (23.50%)	
SB	2 (2.50%)	0 (0.0%)	2 (2.90%)	
SA	1 (1.20%)	0 (0.0%)	1 (1.50%)	

**Table 2:** Image investigation done for SCD patients SCD: sickle cell disease; MCA: middle cerebral artery; PICA: posterior inferior cerebellar artery

Investigations	All patients (n= 81)	Patients with stroke (n =13)	Patients without stroke (n =68)	P-value
Previous transcranial Doppler US done	n (%)			0.538
Yes	6 (7.40%)	0 (0.0%)	6 (8.80%)	
No	75 (92.60%)	13 (100%)	62 (91.20%)	
Transcranial Doppler US results	n (%)			Not applicable
Abnormal	5 (6.20%)	0 (0.0%)	5 (7.40%)	
Normal	1 (1.20%)	0 (0.0%)	1 (1.50%)	
NA	75 (92.60%)	13 (100%)	62 (91.20%)	
CT brain done	n (%)			0.0001
Yes	12 (14.80%)	12 (92.30%)	0 (0.0%)	
No	1 (1.20%)	1 (7.70%)	0 (0.0%)	
NA	68 (84.00%)	0 (0.0%)	68 (100%)	
CT brain results	n (%)			0.0001
Bilateral multiple infarctions	4 (4.90%)	4 (30.80%)	0 (0.0%)	
Left occipitoparietal ischemia/infarction	2 (2.50%)	2 (15.40%)	0 (0.0%)	
Left frontal ischemia	1 (1.20%)	1 (7.70%)	0 (0.0%)	
Left frontal MCA infarction	1 (1.20%)	1 (7.70%)	0 (0.0%)	
Normal	1 (1.20%)	1 (7.70%)	0 (0.0%)	
Right frontoparietal ischemia	1 (1.20%)	1 (7.70%)	0 (0.0%)	
Right PICA infarction	1 (1.20%)	1 (7.70%)	0 (0.0%)	
Watershed infarction	1 (1.20%)	1 (7.70%)	0 (0.0%)	
NA	69 (85.20%)	0 (0.0%)	68 (100%)	
MRI brain done	n (%)			0.0001
Yes	8 (9.90%)	8 (61.50%)	0 (0.0%)	
No	5 (6.20%)	5 (38.50%)	0 (0.0%)	
NA	68 (84.00%)	0 (0.0%)	68 (100.00%)	
MRI brain results	n (%)			0.0001
Bilateral multiple infarctions	3 (3.70%)	3 (23.10%)	0 (0.0%)	
Left occipitoparietal ischemia/infarction	1 (1.20%)	1 (7.70%)	0 (0.0%)	
Left frontal ischemia	1 (1.20%)	1 (7.70%)	0 (0.0%)	
Left frontal MCA infarction	1 (1.20%)	1 (7.70%)	0 (0.0%)	
Left thalamic infarction	1 (1.20%)	1 (7.70%)	0 (0.0%)	
Watershed infarction	1 (1.20%)	1 (7.70%)	0 (0.0%)	
NA	73 (90.10%)	5 (38.50%)	68 (100%)	

**Table 3:** Risk factors of stroke in SCD patients The asterisk sign (*) indicates a statistically significant relationship (p<0.05). SCA: sickle cell anemia; LDH: lactate dehydrogenase

Risk factors	All patients (n= 81)	Patients with stroke (n =13)	Patients without stroke (n =68)	P-value
Family history of SCA	n (%)			0.163
Yes	36 (44.40%)	5 (38.50%)	31 (45.60%)	
No	21 (25.90%)	6 (46.20%)	15 (22.10%)	
Not mention	24 (29.60%)	2 (15.40%)	22 (32.40%)	
Previous history of stroke	n (%)			0.0001*
Yes	5 (6.20%)	4 (30.80%)	1 (1.50%)	
No	39 (48.10%)	8 (61.50%)	31 (45.60%)	
Not mention	37 (45.70%)	1 (7.70%)	36 (52.90%)	
History of previous acute chest syndrome	n (%)			0.269
Yes	28 (34.60%)	3 (23.10%)	25 (36.80%)	
No	53 (65.40%)	10 (76.90%)	43 (63.20%)	
Frequency of prior admissions	3.69±5.21 (0.00-23.00)	2.54±3.33 (0.00-11.00)	3.91±5.48 (0.00-23.00)	0.387
White blood cells count (WBC) K/uL	13.48±5.70 (4.00-29.00)	12.28±5.47 (6.00-24.00)	13.73±5.67 (4.00-29.00)	0.447
Red blood cells count (RBC) M/uL	3.13±0.76 (2.00-5.00)	2.68±0.36 (2.00-3.00)	3.22±0.78 (2.00-5.00)	0.030*
Hemoglobin concentration (g/dl)	8.12±1.18 (5.00-11.00)	7.99±1.16 (6.00-10.00)	8.15±1.20 (5.00-11.00)	0.691
Hematocrit level (Hct) %	24.35±3.69 (17.00-33.00)	23.69±2.86 (17.00-33.00)	24.48±3.85 (17.00-33.00)	0.522
Mean corpuscular volume (MCV) fL	79.44±10.05 (56.00-104.00)	88.75±7.27 (78.00-104.00)	77.54±9.50 (56.00-99.00)	0.0001*
Platelets count (Plt) (K/uL)	371.34±164.29 (133.00-766.00)	381.91±171.19 (133.00-627.00)	369.15±164.42 (133.00-766.00)	0.054
Reticulocytes count (Retic) (%)	11.02±5.84 (2.00-24.00)	12.86±5.38 (6.00-23.00)	10.54±5.92 (2.00-24.00)	0.267
Vitamin D level (nmol/L)	47.76±33.19 (8.00-224.00)	61.98±71.11 (8.00-224.00)	45.48±22.81 (8.00-100.00)	0.194
HbF fraction (%)	13.44±9.81 (1.00-42.00)	10.18±7.14 (3.00-23.00)	14.11±10.19 (1.00-42.00)	0.205
LDH (U/L)	563.52±218.16 (264.00-955.00)	532.85±214.11 (270.00-803.00)	574.25±224.02 (264.00-955.00)	0.674

**Table 4:** Stroke characteristics of SCD patients SCD: sickle cell disease

Characteristics	Patients with stroke (n =13)
Type of stroke	n (%)
Ischemic stroke	12 (92.30%)
Silent cerebral infarction	1 (7.70%)
Stroke symptoms	n (%)
Seizure	7 (53.80%)
Limb weakness	3 (23.08%)
Headache	2 (15.40%)
Asymptomatic	1 (7.70%)
Treatment	n (%)
Exchange transfusion	12 (92.30%)
Antiepileptic	7 (53.80%)
Outcome (Morbidity)	n (%)
Motor sequelae	6 (46.20%)
No sequelae	5 (38.50%)
Convulsion	2 (15.40%)

## Discussion

The most common cause of stroke among children is sickle cell anemia. Sickle cell anemia patients are at 300 fold increased risk for stroke [24]. The prevalence of stroke in this study was 16%, which is comparable with that reported by other studies (6-17%) [25, 26]. In our study, HbSF** **was the most common genotype among stroke patients followed by HbSS, while another study showed that patients with HbSS genotype composed the majority of stroke cases among SCD patients. In the present study, the most significant risk factors for stroke were previous history of stroke, low red blood cells count (RBC), and high mean corpuscular volume (MCV). Previous acute chest syndrome (ACS), anemia, and leukocytosis were statistically insignificant risk factors. In contrast, two studies showed that a history of previous ACS, anemia, and leukocytosis were statistically significant risk factors. These inconsistent results could be due to the small sample size in our study [25, 27].

Among children with sickle cell anemia, the single most important predictor of stroke is transcranial doppler ultrasound (TCD), which is a noninvasive device measuring flow velocities within the intracranial arteries. For 3 years, the annual risk of stroke is 10% as predicted by abnormal TCD results [18, 24]. In our study, none of the stroke patients had previous TCD ultrasound screening. This could be due to the recent implementation of TCD screening for SCD patients in our center. Our study showed that age and gender had insignificant associations with the development of stroke, which is consistent with multiple other retrospective studies [28-30]. Ischemic stroke was the most prevalent type among our study population (92.3%), which is similar to other studies (88-95%) [19, 26, 27].

Regarding stroke symptoms, seizure (53.8%) was the most common presentation, followed by limb weakness (23.08%) and headache (15.40%). Similarly, common presenting symptoms include seizure, limb weakness, or aphasia [19, 29]. In the present study, all overt stroke patients received exchange transfusion (92.30%), and antiepileptic medications were given to those patients presenting with seizures. Similarly, a study reported that a blood transfusion program in the acute stage of stroke and during the following 3 months was beneficial in patients with sickle cell disease [16]. Our study revealed that motor sequelae (46.20%) were the predominant outcomes among stroke patients. In another study, neurologic and neuropsychological sequelae, including motor deficits, were found in 86% of patients [30-31].

## Conclusions

Stroke is a serious complication in pediatric patients with SCD. It affects the quality of life in terms of motor function and the development of seizures. The prevalence of stroke among SCD patients in our study was 16%, which is on the higher side of the international range. Among all risk factors, transcranial Doppler ultrasound screening is the most important predictor of stroke*.* Further research is needed to build a database of stroke and other neurological complications among pediatric patients with SCD in Saudi Arabia.
